# Supplementary material for: Do Support Vector Machines Play a Role in Stratifying Patient Population Based on Cancer Biomarkers?
Source: Arch Proteom Bioinform. Author manuscript; Available in PMC 2021 Nov 12. (PMC7611982)
Supplement: Appendix [file EMS136455-supplement-Appendix.pdf]

Appendix: Lagrange Multipliers

The Lagrangian multiplier method [10] is a technique for finding the local maxima or minima of a function subject to equality constraints. For the general case with the goal of maximising some *f(x)* subject to *g(x)* = 0, the Lagrangian is:

$$L(x, \boldsymbol{\alpha}) = f(x) - \sum_i \alpha_i g_i(x),$$

with the constraint that the partial derivates with respect to both x and α are equal to 0:

$$\frac{\partial L(x, \boldsymbol{\alpha})}{\partial x} = \frac{\partial L(x, \boldsymbol{\alpha})}{\partial \boldsymbol{\alpha}} = 0$$

where the vector $\boldsymbol{\alpha}$ contains the Lagrange multipliers, α_i. Formulating the problem in this way ensures that the original problem is solved: forcing $\frac{\partial L(x,\alpha)}{\partial x}$ to be equal to 0 recovers the 'maximal'/'minimal' constraint, ensuring that the minimal solution is found; forcing $\frac{\partial L(x,\alpha)}{\partial x}$ to be equal to 0 recovers the constraint of $g(x) = 0$, ensuring that all additional constraints are met. In the current case, $\boldsymbol{f(x)} = \frac{1}{2}\boldsymbol{w} \cdot \boldsymbol{w}$ and $g(x) = y_i(\boldsymbol{w}^T\boldsymbol{x}_i + b) - 1$, so the Lagrangian is:

$$\min_{\boldsymbol{w},b} L_P = \frac{1}{2}\boldsymbol{w} \cdot \boldsymbol{w} - \sum_i \alpha_i[y_i(\boldsymbol{w}^T\boldsymbol{x}_i + b) - 1] \quad (5)$$

$$= \frac{1}{2}\boldsymbol{w} \cdot \boldsymbol{w} + \sum_i \alpha_i - \sum_i \alpha_i y_i(\boldsymbol{w}^T\boldsymbol{x}_i + b) \quad (6)$$

Then, by taking partial derivatives with respect to $\boldsymbol{w}$ and b as required:

$$\frac{\partial L_P}{\partial \boldsymbol{w}} = \boldsymbol{w} - \sum_i \alpha_i y_i \boldsymbol{x}_i = 0 \Rightarrow \boldsymbol{w} = \sum_i \alpha_i y_i \boldsymbol{x}_i \quad (7)$$

$$\frac{\partial L_P}{\partial b} = \sum_i \alpha_i y_i = 0 \quad (8)$$

By observing equation 7, it is clear that the answer for the form of $\boldsymbol{w}$ has been obtained: $\boldsymbol{w}$ can be written as a linear combination of all α_i, y_i and $\boldsymbol{x}_i$; so all that remains is to find all α_i. Most of these α_i will be zero, the non-zero α_i terms correspond to the support vectors.

The formulation of equation 6 is known as the **Primal** form of the optimisation problem. Instead of solving this, the **Dual** form of the problem will be solved. In the Primal form of the problem, one is minimising L_P (note the subscript P to represent the Primal) form over $\boldsymbol{w}$ and b with respect to constraints in terms of α_i. In the Dual of the problem, one can maximise over the α_i (the Dual variable) subject to previously obtained constraints in terms of $\boldsymbol{w}$ and b; namely equations 7 and 8. Furthermore, by substituting the values obtained for these constraints into equation 6, the dependencies on $\boldsymbol{w}$ and b can be removed and the final formulation is in terms of α_i only.

The Dual form of the problem is therefore:

$$\max_{\alpha} \; L_D = \frac{1}{2}\sum_i \sum_j \alpha_i \alpha_j y_i y_j (\boldsymbol{x}_i \cdot \boldsymbol{x}_j) + \sum_i \alpha_i$$

$$- \sum_i \alpha_i y_i \boldsymbol{x}_i \sum_j \alpha_j y_j \boldsymbol{x}_j - \sum_i b \alpha_i y_i \quad (9)$$

$$= \sum_i \alpha_i - \frac{1}{2}\sum_i \sum_j \alpha_i \alpha_j y_i y_j (\boldsymbol{x}_i \cdot \boldsymbol{x}_j) \quad (10)$$

$$= \boldsymbol{\alpha}^T \boldsymbol{1} - \frac{1}{2}\boldsymbol{\alpha}^T \boldsymbol{D} \boldsymbol{\alpha}, \quad (11)$$

subject to the constraints $\sum_i \alpha_i y_i = 0$ and $\alpha_i \geq 0$, $i = 1, \ldots, l$. Where **1** is vector of ones of length l i.e. $\boldsymbol{1} = (1, \ldots, l)$ and $\boldsymbol{D}$ is a square, symmetric matrix such that $D_{ij} = y_i y_j (\boldsymbol{x}_i \boldsymbol{x}_j)$.

Equation 11 is the same formulation of the problem obtained in 10, just represented in vector notation. This final form of the optimisation problem is easy to solve using existing quadratic programming methods, meaning that the α_i values can be obtained and $\boldsymbol{w}$ can be calculated. Furthermore, the value for the bias, b, can also be obtained by calculating $b = y_i - \boldsymbol{w}^T \boldsymbol{x}_i$ for each support vector and then averaging over all results.

Finally, with values obtained for $\boldsymbol{w}$ and b, given a novel feature vector **u**, one can obtain its predicted classification based on its observed input variable values by observing on which side of the decision surface it lies; this is achieved by taking the sign (is it positive or negative) of the decision function:

$$sign(\boldsymbol{w}^T \boldsymbol{u} + b) = sign\left(\left[\sum_i \alpha_i y_i (\boldsymbol{x}_i \cdot \boldsymbol{u}_i)\right] + b\right) \quad (12)$$
